# Experiments in Colored Skin Grafting

**Published:** 1877-04

**Authors:** J. H. Wm. Meyer

**Affiliations:** House Physician in Cook County Hospital


REPORTED, March 19, 1877.

---

### EXPERIMENTS IN COLORED SKIN GRAFTING.

By DR. J. H. WM. MEYER.

[House Physician in Cook County Hospital.]

CASE I. Name, Francis Bozee; age, 38; occupation, carpenter; born in France; diagnosis, ulcer of leg. Admitted to Cook Co. Hospital, Sept. 18th, 1876; discharged Jan. 1st, 1877. Recovered.

Last January, patient struck his left shin with the heel of his right boot, breaking the skin. The abrasion increased in size, and finally became a large and deep ulcer.

On admission, we found a nearly circular ulcer, about three inches in diameter, situated over and to the left of the tibia, about the middle of the leg. The edges are ragged and everted; the vicinity of the ulcer much inflamed, and the surface composed of a large slough. Gives a history which indicates a syphilitic origin.

Removed the slough and ordered poultice.

Sept. 29th. The slough having separated, the dressing was changed to ceratum resinæ, and a bandage applied. The ulcer is now four by three inches.

Oct. 10th. The ulcer has undergone no change.

Patient is ordered to keep his bed, the leg is slightly elevated, and three small grafts are placed within half an inch of the outer edge of ulcer.

Oct. 10th, P. M. The surface is suppurating very freely; two of the grafts were washed off with the pus. They were replaced, and the surface left exposed to the air.

11th. Suppuration not so profuse. Grafts keep their places.

12th. The external cuticle of the grafts wrinkled. Cover the ulcer with oiled silk, and wash it twice daily.

13th. External cuticle washes off with the pus.

14th. Grafts are firmly adherent; a tooth-like projection is extending from the outer border of the ulcer toward the central graft.

16th. Projection referred to, has reached and become continuous with the graft. A second projection went through the same process.

23d. Suppuration has much diminished; the grafts and the skin from its edges are covering the ulcer rapidly.

26th. Transplanted a graft from a negro's skin upon the middle of the ulcer.

28th. The black, horny layer looks wrinkled, and is loosening.

29th. This morning it washes off with the pus, leaving the graft looking pink, but not as clear as white skin.

30th. Edges are closing in; grafts are enlarging.

Nov. 3d. Negro's cuticle is growing nicely, but presents, as yet, only a red color.

5th. Fungous granulations around the grafts are lightly brushed with argenti nitratis grs. v. ad aqua ℥i.

13th. Piece of negro's cuticle looks quite black.

31st. Ulcer has all healed over; the black graft remains black.

CASE II. H. Bell, aged 52; occupation, mason; native of Ireland; diagnosis, varicose ulcers of leg. Admitted to Cook County Hospital October 12, 1876; discharged Jan. 2, 1877. Recovered.

Patient has had varicose veins as long as he can recollect. Sixteen years ago a slight abrasion on his left shin was followed by an ulcer of two month's duration. Thiee years ago he had another at the same spot, which was soon healed. Last fall a third ulcer appeared on the outer aspect of the left ankle, and another on the inner side.

On admission, we find large, ill-conditioned ulcers around the left ankle. Applied ceratum resinæ on all but the largest, upon which two grafts from a negro's skin were placed, and covered with oiled silk.

Oct. 14th. Both grafts washed off with the pus, but were replaced.

Oct. 15th. Grafts adherent; horny layer wrinkled.

19th. Horny layer of grafts has fallen off; true skin is adherent; looks pink.

Ulcer closing in towards the grafts; very little suppuration.

Oct. 22d. During the night one of the grafts was pulled off by the clothes; the other is firmly adherent; looks blackish.

30th. Edge of ulcer has become continuous with the graft, which has grown about three-fourths of an inch. The original graft is as black as the negro's skin from which it was taken, and fades out towards the border. About three-fourths of an inch from the centre of the graft, the skin is as white as that of the patient.

